# The H50Q Mutation Induces a 10-fold Decrease in the Solubility of α-Synuclein*

**DOI:** 10.1074/jbc.M114.610527

**Published:** 2014-12-10

**Authors:** Riccardo Porcari, Christos Proukakis, Christopher A. Waudby, Benedetta Bolognesi, P. Patrizia Mangione, Jack F. S. Paton, Stephen Mullin, Lisa D. Cabrita, Amanda Penco, Annalisa Relini, Guglielmo Verona, Michele Vendruscolo, Monica Stoppini, Gian Gaetano Tartaglia, Carlo Camilloni, John Christodoulou, Anthony H. V. Schapira, Vittorio Bellotti

**Affiliations:** From the ‡ Wolfson Drug Discovery Unit, Centre for Amyloidosis and Acute Phase Proteins, Division of Medicine, and; the § Department of Clinical Neuroscience, Institute of Neurology, University College London, London NW3 2PF, United Kingdom,; the ‖ Centre for Genomic Regulation and University Pompeu Fabra, 08003 Barcelona, Spain,; the ** Department of Molecular Medicine, Institute of Biochemistry, University of Pavia, 27100 Pavia, Italy,; the ¶ Department of Structural and Molecular Biology, University College London, London WC1E 6BT, United Kingdom,; the §§ Department of Chemistry, University of Cambridge, Cambridge CB2 1EW, United Kingdom, and; the ‡‡ Department of Physics, University of Genoa, 16146 Genoa, Italy

**Keywords:** alpha-Synuclein (a-synuclein), Amyloid, Fibril, Parkinson Disease, Protein Aggregation, Aggregation Propensity, Fibrils Thermodynamic Stability, Polyproline II Structure

## Abstract

The conversion of α-synuclein from its intrinsically disordered monomeric state into the fibrillar cross-β aggregates characteristically present in Lewy bodies is largely unknown. The investigation of α-synuclein variants causative of familial forms of Parkinson disease can provide unique insights into the conditions that promote or inhibit aggregate formation. It has been shown recently that a newly identified pathogenic mutation of α-synuclein, H50Q, aggregates faster than the wild-type. We investigate here its aggregation propensity by using a sequence-based prediction algorithm, NMR chemical shift analysis of secondary structure populations in the monomeric state, and determination of thermodynamic stability of the fibrils. Our data show that the H50Q mutation induces only a small increment in polyproline II structure around the site of the mutation and a slight increase in the overall aggregation propensity. We also find, however, that the H50Q mutation strongly stabilizes α-synuclein fibrils by 5.0 ± 1.0 kJ mol^−1^, thus increasing the supersaturation of monomeric α-synuclein within the cell, and strongly favors its aggregation process. We further show that wild-type α-synuclein can decelerate the aggregation kinetics of the H50Q variant in a dose-dependent manner when coaggregating with it. These last findings suggest that the precise balance of α-synuclein synthesized from the wild-type and mutant alleles may influence the natural history and heterogeneous clinical phenotype of Parkinson disease.

## Teaser

**Background:** The basis of the pathogenicity of the H50Q variant α-synuclein is unknown.

**Results:** The critical concentration of α-synuclein is decreased by 10-fold by the H50Q mutation, and its aggregation is modulated by the wild-type isoform.

**Conclusion:** Key effects of the H50Q mutation on the aggregation of α-synuclein can be quantified.

**Significance:** Our data provide insights into the mechanism of Lewy body formation *in vivo*.

## Introduction

α-Synuclein (α-Syn)^4^ is central to the pathogenesis of Parkinson disease (1, 2). Mutations and multiplications of the encoding *SNCA* gene are associated with familial Parkinson disease and polymorphisms with an increased risk of developing sporadic disease (3–7). Monomers of α-Syn have been shown to be capable of forming soluble oligomers and amyloid fibrils (8–10), which are the major component of intraneuronal Lewy bodies, the pathological hallmark of Parkinson disease (11, 12). The molecular basis of the transition of α-Syn from its intrinsically disordered monomeric state (13–15) into cross-β fibrillar assemblies is largely unknown. Future progress is expected from the complementary synergy of two approaches: continuous optimization of algorithms suitable for predicting the aggregation propensity of the protein and the production of experimental models of α-Syn fibrillogenesis mimicking the physiological environment. Particularly informative is the investigation of natural pathogenic mutations of α-Syn associated with misfolding and aggregation.

The recently identified H50Q α-Syn mutation (16, 17) was shown to aggregate faster *in vitro* compared with the WT counterpart (18–21). Here we compare sequence-based predictions of aggregation propensity (22, 23) with further experimental observations of aggregation behavior in the WT and H50Q α-Syn. It is worth noting that in heterozygous carriers, as in the patients with the H50Q mutation, half of the expressed α-Syn is expected to be WT. Although current aggregation propensity algorithms are unable to predict the effect of mixtures of α-Syn variants upon the aggregation process, our experimental data show that the WT protein attenuates the aggregation kinetics of the variant in a dose-dependent manner. However, the molecular explanation of the effect of H50Q and other mutations on the aggregation propensity remains an unresolved issue.

To investigate further the causes of the increased aggregation propensity of the H50Q variant, we characterize the monomeric ensembles of both WT and H50Q α-Syn using NMR spectroscopy. In previous reports, comparative spectroscopic analyses have revealed broadly similar structural ensembles in the WT and H50Q variants, with a small number of chemical shift changes around the site of the mutation (18–20) and, in some cases, additional perturbations in the C-terminal region (18, 19). Here we carry out a comprehensive chemical shift-based analysis of WT and H50Q α-Syn, using the δ2D algorithm (24) to scrutinize changes in the residual secondary structure of the variant.

By recognizing that to understand the effect of a mutation it is important to consider the potential effects on the fibrillar end products of the aggregation process, we use chemical denaturation of fibrils to determine the free energy of elongation. These measurements provide access to the critical concentration, *i.e.* the concentration above which fibrils are thermodynamically the most stable state (25–27). Therefore, we characterize the relative stabilities of fibrils formed from WT α-Syn and the H50Q disease-associated variant, finding that the mutation has a major impact on the solubility of the protein.

## EXPERIMENTAL PROCEDURES

### 

#### 

##### Aggregation Propensities with Solvent Exposure Corrections

**FIGURE 1.**
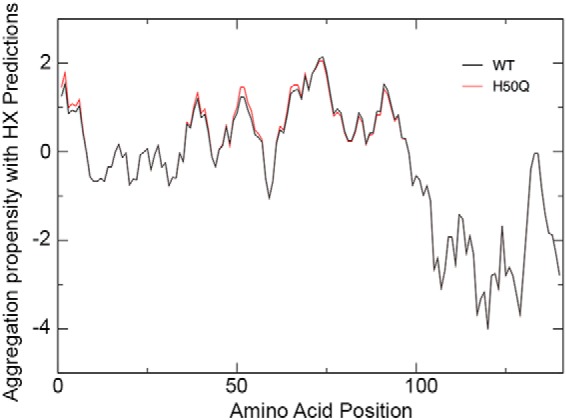
**Prediction of aggregation propensity of WT and H50Q α-Syn.** Shown are CamP-corrected aggregation profiles of WT (*black*) and H50Q α-Syn (*red*) as calculated with the Zyggregator method (23). *HX*, hydrogen exchange.

The intrinsic aggregation propensity profile is calculated using the position-dependent score *P*_*i*_^agg^. For a given residue *i*, the score is calculated as follows,

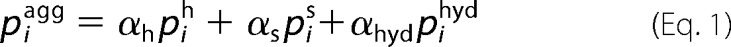
 where *p*_*i*_^h^ and *p*_*i*_^s^ are the propensities for α-helix and β-sheet formation, respectively, and *p*_*i*_^hyd^ is the hydrophobicity (23). The values are combined to provide a score, *Z*_*i*_^agg^, that describes the intrinsic propensity for aggregation for the whole amino acid sequence (23, 28),

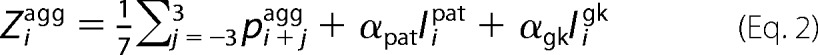
 where *I*_*i*_^pat^ is the term that takes into account the presence of specific patterns of alternating hydrophobic and hydrophilic residues (29), and *I*_*i*_^gk^ is the term that takes into account the gatekeeping effect of individual charges (23).

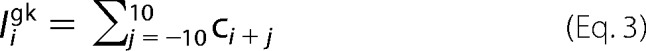
 By combining the predictions of the intrinsic aggregation propensity profiles with those for the solvent exposure of protein regions, it is possible to account for the influence of transient structure formation on the aggregation propensities. The aggregation propensity profile is defined by modulating the intrinsic aggregation propensity profile with the CamP score, denoted as *P_i_*, predicting the local structural stability at that position (30). The propensity of exposed regions to promote aggregation can be expressed as follows,

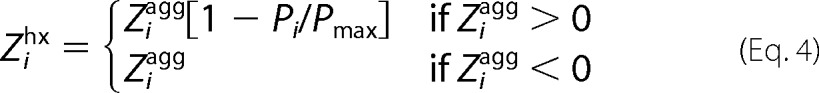
 where *P*_max_ = 15 is a normalization constant (23). The number of Fourier coefficients employed to obtain the results in Fig. 1 is 7 (23, 30).

##### Protein Expression and Purification

Recombinant WT and H50Q variant α-Syn were expressed and purified as previously described (31). For H50Q α-Syn, the QuikChange site-directed mutagenesis kit (Stratagene) was used with the primer sequence GAGGGAGTGGTGCAAGGTGTGGCAACAGTG containing the underlined codon for glutamine at position 50.

##### Kinetics of Fibrillogenesis

Samples of recombinant WT and H50Q α-Syn, 100 μl at different concentrations (5, 10, 30, 50, 70, and 100 μm, respectively) in PBS, pH 7.4, containing 10 μm thioflavin T (ThT) (32), were incubated at 37 °C in Costar 96-well black wall plates sealed with sealing film (4titude Gas Permeable Moisture Barrier Seal) and subjected to 900 rpm double orbital shaking. Bottom fluorescence was recorded at 15-min intervals (FLUOstar Omega, BMG LABTECH). Time courses of aggregation were fitted to a sigmoidal model, as *y* = *y*_0_ + (*y*_max_ − *y*_0_)/(1 + exp[−*k*_app_(*t* − *t*_½_)]) using KaleidaGraph 4.0 (Synergy Software, Reading, PA), where *y*_0_ and *y*_max_ are the initial and maximum ThT fluorescence, respectively; *k*_app_ is the apparent rate constant, and lag time was defined as *t*_½_ − 2/*k*_app_ (18). Experiments were conducted in triplicate in three independent experiments. Samples containing the ThT positive material were further analyzed by electron microscopy. Further aggregation time courses were performed using mixtures of WT/H50Q at 0:1, 1:5, 1:2, 1:1, 2:1, 5:1, and 1:0 molar ratios, respectively, keeping the total protein concentration at 70 μm.

##### Electron Microscopy

Formvar-coated copper EM grids were placed coated side down onto each sample and incubated for 2 min before blotting with filter paper to remove excess solvent and staining with 2% (w/v) uranyl acetate for 2 min. After further blotting and drying in air, transmission electron microscope (CM120) images were obtained at 80 keV.

##### Amyloid Fibril Preparation

A scaled up method was developed to prepare larger quantities of fibrils for further characterization. Briefly, solutions of WT and H50Q α-Syn at 5 mg/ml in PBS, pH 7.4, were stirred at 1500 rpm for 72 h at 37 °C. Finally, fibrillar aggregates were quantified by assessment of the monomer left in the supernatant considering that the molar absorptivity is 5960 m^−1^ cm^−1^ for both WT and variant α-Syn.

##### Equilibrium Unfolding of WT and H50Q α-Syn Fibrils

Fibrils (0.5 mg/ml) in PBS, pH 7.4, were incubated with increasing concentrations of guanidine HCl (Merck) from 0 to 5.5 m. Samples were thoroughly mixed by vortexing and incubated at room temperature for 72 h prior to centrifugation in a Beckman Optima TL ultracentrifuge at 135,000 × *g* for 45 min. The incubation time was experimentally verified to be sufficient for the samples to reach equilibrium, and the monomer concentration in the supernatant was quantified as previously described (25). Experiments were conducted in triplicate. Size exclusion chromatography of WT and H50Q fibrils after denaturation and ultracentrifugation was performed using a Superdex 200 column on the ÄKTA Explorer apparatus (GE Healthcare). The column was equilibrated and eluted at 0.5 ml/min with PBS buffer, pH 7.4. WT and H50Q α-Syn at 0.5 mg/ml in PBS were also run as control. The fraction of soluble monomeric α-Syn over the total concentration was plotted with denaturant concentration for further analysis.

##### Determination of Thermodynamic Stability Parameters

The equilibrium unfolding curves of α-Syn fibrils were analyzed using a linear polymerization model (25, 33, 34) [F*_i_*_−1_] + [M] ⇄ [F*_i_*], in which [M] and [F*_i_*] represent the concentration of monomers and fibrillar aggregates of size *i*, respectively, with the equilibrium constant *K* = *c*^0^[F*_i_*]/[F*_i_*_−1_][M], where *c*^0^ is the standard concentration 1 mol liter^−1^. Based on this model the fraction of monomeric α-Syn over the total protein concentration, [M]/[M_T_], can be expressed as follows.

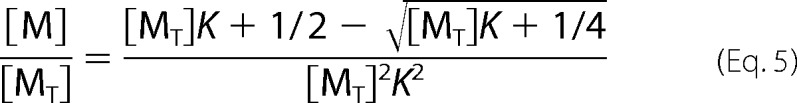
 The equilibrium constant *K* can also be expressed as *K* = exp(−Δ*G*_el_/*RT*), in which Δ*G*_el_ is the free energy of elongation, *R* is the gas constant, and *T* is the absolute temperature. In the presence of chemical denaturants, *i.e.* guanidine HCl, Δ*G*_el_ is linearly dependent on the concentration of denaturant, [D], according to Δ*G*_el_ = *m*[D]+ Δ*G*_el_^0^, where *m* is a cooperativity coefficient, and Δ*G*_el_^0^ is the free energy of elongation in the absence of denaturants (25). The experimental data of the equilibrium unfolding of WT and H50Q α-Syn fibrils were fitted to Equation 5 to obtain the main thermodynamic parameters using KaleidaGraph 4.0 (Synergy Software). Values of midpoint denaturant concentration, [D]_50%_, and the critical concentration, *c*_crit_ = *c*^0^ exp(Δ*G*_el_/*RT*), were also calculated. All measurements are reported as means ± S.D. of three independent experiments.

##### Atomic Force Microscopy

After a 500-fold dilution, 10 μl of α-Syn fibrils were finally deposited on freshly cleaved mica and dried under mild vacuum. Tapping mode AFM images were acquired in air using a Dimension 3100 Scanning Probe Microscope and a Multimode Scanning Probe Microscope (Digital Instruments, Bruker). Single beam uncoated silicon cantilevers (type OMCL-AC160TS; Olympus) were used. The drive frequency was between 290 and 310 kHz; the scan rate was between 0.4 and 0.5 Hz. Fibril height was measured from the cross-section height of topographic AFM images.

##### NMR Spectroscopy

NMR data were acquired at 283 K using a 700 MHz Bruker Avance III NMR spectrometer equipped with a TXI cryoprobe. Uniformly ^15^N/^13^C-labeled samples of WT and H50Q α-Syn were prepared as previously described (35), at concentrations of 500 and 700 μm, respectively, in 10 mm sodium phosphate buffer, 100 mm NaCl, pH 7.5, 5% D_2_O, 0.01% NaN_3_, 0.001% dimethyl-silapentane-sulfonate. ^1^H,^15^N heteronuclear single quantum coherence spectroscopy, BEST-HNCO, BEST-iHNCO, BEST-HNCOCACB, BEST-HNCACB, HA(CO)NH, and HNHA experiments were recorded to assign the backbone HN, N, C, CA, CB, and HA chemical shifts of both WT and H50Q α-Syn (36, 37). All spectra were processed using nmrPipe (38) and Collaborative Computing Project for NMR analysis (39) and were referenced using the internal dimethyl-silapentane-sulfonate chemical shift (40). The measured chemical shift values of WT and H50Q α-Syn have been deposited in the Biological Magnetic Resonance Data Bank (41). Secondary structure populations were calculated from chemical shifts using the δ2D web server (version 1.2) (24).

## RESULTS

### 

#### 

##### Prediction of the Aggregation Propensity

We studied the aggregation propensity of H50Q α-Syn using the Zyggregator method (23, 42). The effects of the His to Gln mutation on the conformational fluctuations of α-Syn were considered via the CamP algorithm (30), which provides a prediction of transient structure formation and solvent accessibility in different regions of the protein. By combining solvent exposure contributions and aggregation profiles (23), we found that the overall aggregation propensity of the H50Q variant is increased by ∼5% compared with WT α-Syn, from 0.84 to 0.88 (Fig. 1). More specifically, we observe an increase in aggregation propensity for residues 35–47 (as the area below the curve increases from 12 to 15) and residues 61–96 (as the area below the curve increases from 33 to 34). Importantly, glutamine 50 is predicted to allow exposure of the amino acid stretches around this position, which increases the ability to establish long range interactions and promote aggregation. This analysis is in agreement with predictions of residue burial (43) and accessibility (44) calculated by independent methods.

##### Kinetic Analysis of the Aggregation Process

**FIGURE 2.**
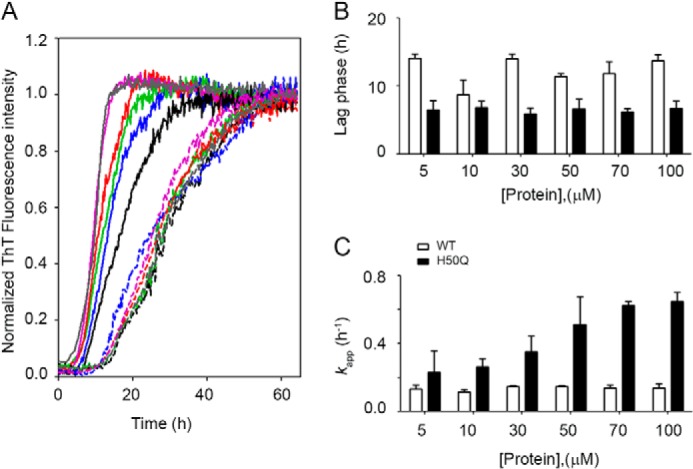
**Kinetics of fibrillogenesis.** *A*, normalized fluorescence data of WT (*dashed lines*) and H50Q (*solid lines*) α-Syn at six different concentrations: 100 μm (*gray*), 70 μm (*purple*), 50 μm (*red*), 30 μm (*green*), 10 μm (*blue*), and 5 μm (*black*), respectively. *B*, effect of initial protein concentration on the duration of the lag phase in the aggregation of WT (*open bars*) and H50Q α-Syn (*solid bars*), respectively. *C*, dependence of *k*_app_, apparent growth rate of fibrils, over initial monomer concentration for WT and H50Q α-Syn. The *bars* represent means ± S.D. of four independent experiments.

Both WT and H50Q α-Syn aggregation time courses were measured under physiological conditions and constant double orbital agitation over the concentration range of 5–100 μm. Analysis of normalized ThT fluorescence data (Fig. 2) shows that both proteins form amyloid fibrils following a concentration-independent lag phase (Fig. 2*B*), which is consistently shorter for the H50Q variant of ∼2-fold at each concentration except at 10 μm and, overall, in agreement with previous results (18). Apparent growth rates (*k*_app_) were quantified from the same ThT data (Fig. 2*A*) and confirm that H50Q α-Syn aggregates faster than WT (20) with the highest differences at 70 and 100 μm where H50Q α-Syn *k*_app_ exhibits a rate of ∼0.6 h^−1^ compared with 0.1 h^−1^ for WT α-Syn.

**FIGURE 3.**
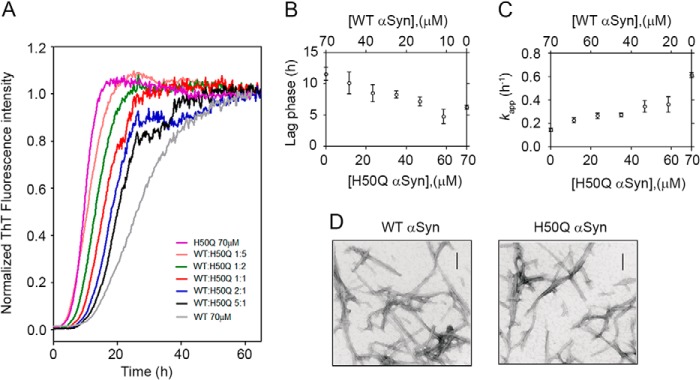
**Mutual effect of WT and H50Q α-Syn on the aggregation kinetics.** *A*, normalized data of mixtures of WT/H50Q α-Syn at 1:5 (*orange*), 1:2 (*green*), 1:1 (*red*), 2:1 (*blue*), and 5:1 (*black*) molar ratios, respectively. *Curves* at 70 μm of WT (*gray*) and H50Q (*purple*) were also included. *B*, effect of increasing concentration of H50Q (or WT) α-Syn on the lag time of aggregation. *C*, effect of increasing concentration of H50Q (or WT) α-Syn on the *k*_app_ of aggregation. Means ± S.D. of at least three independent experiments are shown. *D*, microscopic analysis of *in vitro* fibrils of WT and H50Q α-Syn. *Scale bars*, 100 nm.

Because patients carrying the H50Q mutation are expected to express both WT and variant protein, we investigated further the aggregation kinetics of a mixture of the two species. Different proportions of WT and H50Q α-Syn were prepared, keeping constant the total concentration of α-Syn at 70 μm (Fig. 3). A progressive prolongation of the lag phase is directly dependent on the relative proportion of the WT over the variant (Fig. 3*B*), and on the contrary, the WT aggregates faster by increasing the concentration of the variant (Fig. 3*C*).

##### Thermodynamic Analysis of α-Syn Fibrils

**FIGURE 4.**
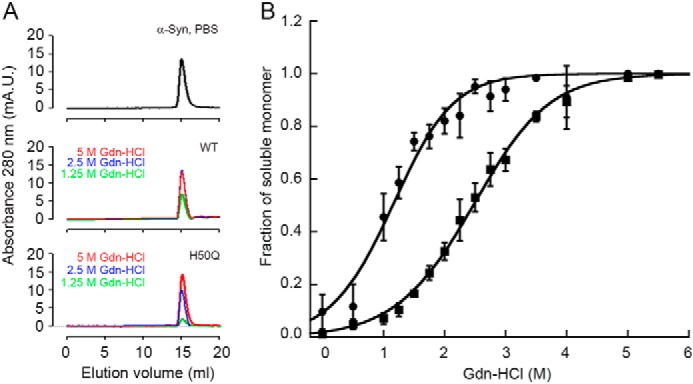
**Thermodynamic stability of *in vitro* fibrils formed by WT and H50Q α-Syn.** *A*, size exclusion profile of ultracentrifuged samples of WT and H50Q α-Syn fibrils after denaturation with guanidine HCl. Representative curves at 5, 2.5, and 1.25 m guanidine HCl, respectively, show a single peak eluting at the same retention time as the native monomer (either WT or H50Q α-Syn) in PBS. Both the two isoforms show the same pattern when applied to a Superdex 200 column equilibrated and eluted with PBS at 0.5 ml/min. *mA.U.*, milli absorbance units. *B*, the proportion of monomer released from WT (*circles*) and H50Q (*squares*) α-Syn fibrils over the total protein concentration at increasing guanidine HCl (*Gdn-HCl*) concentrations was analyzed with Equation 1 following the linear polymerization model as described under “Experimental Procedures.” *Curves* shown as mean (S.D.) of three independent experiments.

Although when examined under the electron microscope, our fibrillar aggregates do not exhibit major differences (Fig. 3*D*), we found clear evidence that H50Q α-Syn fibrils are more resistant to denaturation than WT. We titrated WT and H50Q α-Syn fibrils with guanidine HCl and analyzed the material in solution after 72 h of incubation at each concentration of denaturant. Size exclusion chromatography showed a single peak eluting from the column at the same retention time as the native monomer (Fig. 4*A*). The fractions of the quantified soluble monomer over the total protein concentration were fitted with the linear polymerization model, as described under “Experimental Procedures.” These results clearly show that the fibrils formed by WT α-Syn are significantly less stable than those formed by H50Q α-Syn (Fig. 4*B*), with a midpoint concentration of guanidine HCl decreasing from 2.4 ± 0.1 m for the variant to 1.2 ± 0.1 m for WT and a difference in the free energy of elongation in the absence of denaturant, ΔΔ*G*_el_^0^ = Δ*G*_el_^0^_H50Q_ − Δ*G*_el_^0^_WT_, of −5.0 ± 1.0 kJ mol^−1^ (Table 1). This difference corresponds to a significant reduction in the critical concentration, *i.e.* in the solubility, from 4.1 ± 0.1 μm for WT α-Syn to 0.6 ± 0.2 μm for the H50Q variant.

**TABLE 1:** **Thermodynamic parameters of guanidine HCl induced unfolding of α-synuclein fibrils** All of the values in the table are means ± S.D. of three independent experiments. The values are as follows: [D]_50%_ (m), midpoint concentration of guanidine HCl; Δ*G*_el_^0^ (kJ mol^−1^), free energy of association in absence of denaturant; *m* (kJ mol^−1^ m^−1^), dependence of Δ*G*_el_ on denaturant concentration; *c*_c_, critical concentration (μm).

α-Synuclein	[*D*]_50%_	Δ*G*_el_^0^	*m*	*c*_c_
	*m*	*kJ mol*^−*1*^	*kJ mol*^−*1*^ *m*^−*1*^	μ*m*
WT	1.2 ± 0.1	−30.8 ± 0.1	5.6 ± 0.5	4.1 ± 0.1
H50Q	2.4 ± 0.1	−35.4 ± 0.9	4.5 ± 0.3	0.6 ± 0.2

**FIGURE 5.**
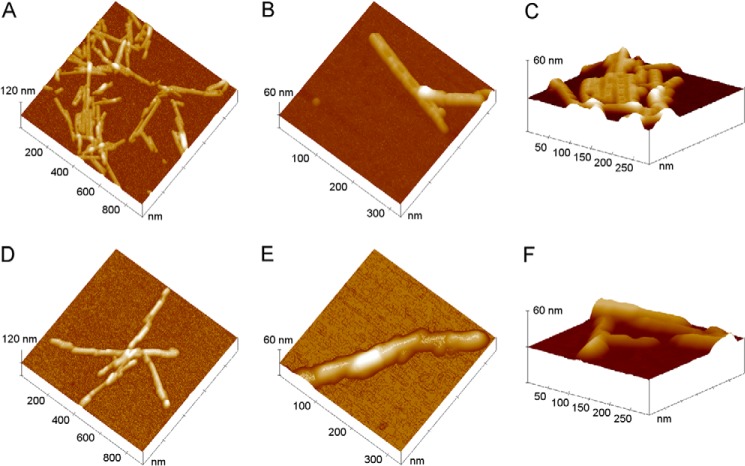
**AFM analysis of *in vitro* fibrils formed by WT and H50Q α-Syn.** *A–C*, surface plots of topographic AFM images of H50Q α-Syn fibrils. *D–F*, WT α-Syn fibrils.

Accurate AFM measurements reveal that WT and H50Q fibrils do in fact exhibit different structural details (Fig. 5). Although in both cases fibrils are short and straight, with typical lengths ranging from 100 to 800 nm, fibrils formed by H50Q α-Syn mainly consist of rods with relatively uniform width, in some cases exhibiting a periodic structure (Fig. 5, *A–C*). The structured fibril profile appears to be symmetric and therefore compatible with an even number of constituent subunits, in agreement with previous findings (18), although our AFM images suggest that these fibrils are formed by apposition of two incomplete rods rather than from intertwining of protofilaments. On the contrary, there is no evidence of periodicity in the WT α-Syn fibrils. The latter result from the assembly of different units slightly staggered along the fibril axis, giving rise to an irregular variation of the fibril width along the fibril (Fig. 5, *D–F*). The structural differences between WT and H50Q α-Syn fibrils are also reflected in the fibril heights, which are 5.8 ± 0.1 nm for H50Q α-Syn and 8.3 ± 0.2 nm for the WT species, respectively. Overall, the structural features observed from AFM data suggest a more ordered and compact packing of the protein in H50Q α-Syn fibrils compared with the WT species. These findings may be correlated with the different fibril stability observed for the variant and WT α-Syn.

##### NMR Analysis of Residual Secondary Structure in the Disordered State

**FIGURE 6.**
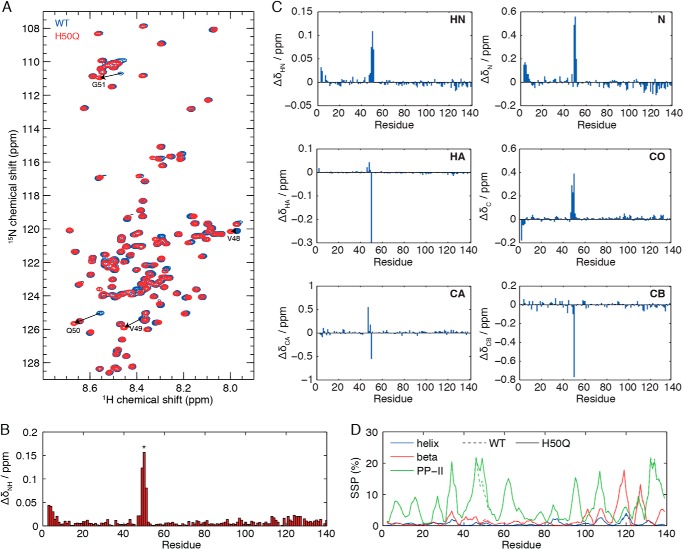
**NMR characterization of residual structure in monomeric H50Q.** *A*, overlay of ^1^H,^15^N heteronuclear single quantum coherence spectroscopy spectra of WT (*blue*) and H50Q (*red*) α-Syn. *Arrows* highlight cross-peaks that exhibit large chemical shift changes between the WT and the variant. *B*, amide chemical shift differences between WT and H50Q α-Syn, calculated as the weighted combination Δδ_NH_ = (Δδ_H_^2^ + (Δδ_N_/5)^2^)^½^. Residue 50 is highlighted with an *asterisk* (*). *C*, chemical shift perturbations of HN, N, HA, C, CA, and CB resonances in H50Q. Glycine HA chemical shifts are reported as the averages of both chemical shifts. *D*, secondary structure populations of WT (*dashed lines*) and H50Q (*solid lines*) calculated from backbone chemical shift data using the δ2D algorithm (24).

Two-dimensional ^1^H,^15^N heteronuclear single quantum coherence spectroscopy correlation spectra of α-Syn and H50Q α-Syn closely overlay (Fig. 6*A*), thus indicating that both proteins populate highly heterogeneous conformational ensembles. Small amide chemical shift perturbations are found close to the site of mutation (Fig. 6*B*), localized to residues Val^48^–Gly^51^. NMR chemical shifts are highly sensitive probes of local structure, and therefore to investigate perturbations to residual secondary structure in the variant, we measured complete sets of HN, N, C, CA, CB, and HA backbone chemical shifts in both WT and H50Q α-Syn. Chemical shift perturbations in all spins were small and restricted to the vicinity of the mutation (Fig. 6*C*). We then used the δ2D algorithm (24) to calculate and compare secondary structure populations in the WT and H50Q variant (Fig. 6*D*). Very few residual α-helical populations are observed in either species, but polyproline II and β-sheet populations of up to 20% are found across the length of the sequence. A 7% increase in polyproline II population is observed around the site of the mutation in H50Q, but otherwise the perturbations from the WT are very small.

## DISCUSSION

The aggregation kinetics data discussed above are consistent with previous reports that the H50Q mutation in the α-Syn sequence is associated with an increased aggregation propensity (18–21). This mutation was independently reported in an apparently sporadic British case and in a familial Canadian case with British ancestry, with evidence of a founder effect (16, 17).

Oligomerization and aggregation of α-Syn have long been considered to be crucial in the pathogenesis of Parkinson disease, although the precise toxic species remains a matter of debate (45). Previously reported missense mutations have accordingly generally been shown to enhance this process. However, the picture is far from clear, and the mechanism by which mutations modulate aggregation kinetics remains unsolved. In this study, we have shown that the WT protein, also expected to be expressed in all heterozygous patients, can modulate the aggregation kinetics of the mixture of WT and H50Q α-Syn. These findings suggest that availability of data on the intracellular molar ratio between variant and WT protein in these patients would be crucial to elucidate the natural history of the disease and interpret some unexplained heterogeneous clinical features.

In patients with the A53T mutation, there is evidence that the expression levels of the WT and mutant alleles may not always be equal (46, 47). It is unknown whether the H50Q mutation is expressed at the same levels as WT α-Syn *in vivo*. Interestingly, H50Q falls into a region that has been proposed to regulate α-Syn expression through a negative feedback loop between the protein and its own mRNA (48). H50Q is predicted to reduce the regulatory potential of α-Syn, which suggests an increase in protein expression and an enhanced tendency to aggregate.

**FIGURE 7.**
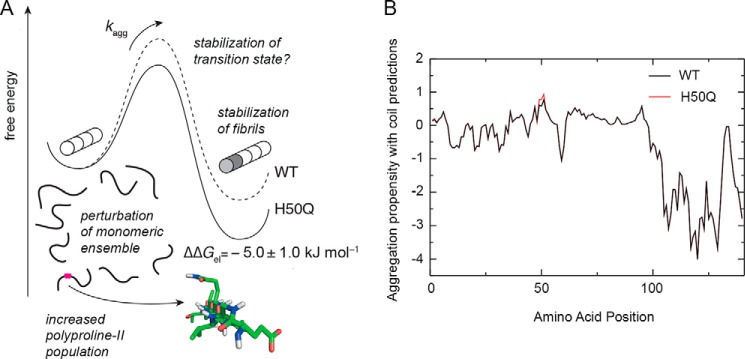
**Summary of factors affecting the aggregation propensity of WT and H50Q α-Syn.** *A*, schematic free energy landscape for the elongation of WT and H50Q fibrils, illustrating the observed stabilization of H50Q variant fibrils and the potential stabilization of the transition state. Changes to secondary structure populations within the monomeric ensemble are also indicated, and a fragment of α-Syn H50Q (residues *x-y*) structure is also shown, modeled in a polyproline II conformation. *B*, measured changes in residual secondary structure can potentially be used to improve the accuracy of the predicted aggregation profiles of WT (*black*) and H50Q α-Syn (*red*) by combining the Zyggregator profiles with the β and polyproline II secondary structure populations, *P*^beta^ and *P*^PP^, calculated from experimental chemical shifts (Fig. 6*D*): *Z*_*i*_^cs^ = *Z*_*i*_^agg^(*P*_*i*_^beta^ + *P*_*i*_^PP^)/(*P*^beta^ + *P*^PP^)_max_.

Although the effect of the mutation on the aggregation kinetics is generally observed (18–21), the structural basis of this pathogenic property has hitherto not been fully explained because of the intrinsic difficulty of singling out abnormally structured species in an ensemble of different conformers. A comparison of WT, H50Q, and H50R mutations indicated that a positive charge at residue 50 suppresses aggregation; however, the aggregation of H50A and H50D variants was slower than H50Q, indicating that aggregation behavior is not solely determined by electrostatic effects (19). A powerful technique to investigate the conformation of α-Syn monomers in solution is NMR spectroscopy, and at least three studies of the H50Q variant have recently been reported (18–20). The extent and magnitude of amide chemical shift perturbations (Δδ_NH_ up to 0.15) that we observe here around the site of the mutation (Fig. 7*B*) are very similar to these other reports. However, in one case very large chemical shift perturbations (Δδ_NH_ ∼0.5) were also observed for residues Asp^135^, Tyr^136^, and Glu^137^ in the C terminus of the protein (19). We have not observed evidence of these chemical shift changes in any of our experiments, despite identical experimental conditions (10 mm sodium phosphate, 100 mm NaCl, pH 7.4, 283 K). Khalaf *et al.* (20) suggested that the C-terminal chemical shift perturbations observed by Chi *et al.* (19) may arise from metal ion contamination; we also note that multiple cross-peaks can be observed for some C-terminal resonances (*e.g.* Ala^140^) in the spectra of Chi *et al.* (19), raising the additional possibility of degradation or other covalent modification. We have not observed such additional resonances in any of our own spectra.

Ghosh *et al.* (18) also reported small chemical shift perturbations in the C-terminal region between residues 113 and 135, at pH 6 in the absence of salt. As noted by Khalaf *et al.* (20), chemical shifts in this region are highly sensitive to the electrostatic environment and the ionic strength. NMR titration experiments have shown that His^50^ has a p*K_a_* of 6.5–6.8 (dependent on ionic strength) (49). Therefore at pH 6, the H50Q mutation eliminates a positive charge, whereas at pH 6.8 (20) or physiological pH 7.4, as in our study, the charge on His^50^ is reduced and perturbations in the C terminus arising from the H50Q mutation are also diminished. Khalaf *et al.* (20) investigated the effect of the H50Q mutation on paramagnetic relaxation enhancement effects arising from a nitroxide spin-label positioned at residue 20, using an engineered E20C variant. PREs provide a sensitive probe of transient long range tertiary structure in the disordered state and previously uncovered a weak interaction between the C-terminal region of α-Syn with the N-terminal and the 61–95 non-amyloid-β component regions (50, 51). However, the H50Q variant was not observed to perturb these long range PREs, indicating that the residual tertiary structure of the variant is indistinguishable from that of the WT (20). Khalaf *et al.* (20) also conducted a preliminary investigation of the residual secondary structure of WT and H50Q α-Syn by comparison of Cα secondary chemical shifts in a solution of bacterial lysate and, as measured in detail in the present study, no significant differences were detected.

NMR chemical shift analysis represents an effective and sensitive tool for understanding residual secondary structure in disordered proteins, with the δ2D method having an accuracy of ∼2% for the detection of changes in secondary structure populations of disordered proteins (24). We have previously applied this method (24) to characterize the secondary structure populations of α-Syn expressed within living cells, using in-cell NMR spectroscopy (52, 53), and small decreases in β and polyproline II populations (<5%) were observed across the sequence, but otherwise the protein was found to have the same disordered structure as in dilute aqueous solutions. The comprehensive analysis of secondary structure populations in WT and H50Q α-Syn that we have presented here shows only a small increase (<7%) in the polyproline II population, specific to the site of the mutation (Fig. 6*D*). By comparison, a similar study of the A53T variant observed a 10% increase in the population of β-structure around the site of the mutation (54). An analysis of perturbations to secondary structure populations measured systematically for a series of α-Syn variants (55) highlighted that the population of β-structure correlated strongly (*r* = 0.93) to the aggregation propensity (54). However, much weaker correlations were observed with the formation of α-helical (*r* = 0.24) or polyproline II (*r* = 0.19) structure. Given that only eight variants were analyzed (55), the specific values of these coefficients of correlation should be considered with caution, and further work will be needed to firmly establish the association between the changes in the α-helical or polyproline II populations and the changes in aggregation rate.

In this context, it is notable that both β and polyproline II secondary structures are suitable for the formation of intermolecular hydrogen bonds and could thus promote aggregation. These structured elements, if exposed, may be able to promote more stable initial interactions than those established by coil regions, leading to increased aggregation potential (Fig. 7). Incorporating experimental measurements of β and polyproline II populations into calculations of aggregation propensity may in the future be used to improve the accuracy of these predictions. Such calculations are illustrated in Fig. 7, using secondary structure populations determined by NMR (Fig. 6*D*) together with a scoring function with a form similar to that used to correct for solvent exposure (Equation 4).

In addition to the perturbations to secondary structure in the monomeric ensembles of WT and H50Q α-Syn, we have also identified a significant increase in the thermodynamic stability of H50Q α-Syn fibrils compared with the WT species, ΔΔ*G*_el_ = −5.0 ± 1.0 kJ mol^−1^ (Fig. 7). This finding might be consistent with some morphological differences revealed by EM (18) and our AFM analyses, but the difficulty in determining the structure of amyloid fibrils precludes a detailed structural interpretation of these differences. However, structural studies of amyloid fibrils are rapidly improving in resolution and scale (56), and several solid state NMR models of the α-Syn fibril core indicate that His^50^ is close to the edge of a β-strand or in an adjacent loop region (57–59). The increased stability of H50Q fibrils may therefore arise from a stabilization or extension of this β-strand within the fibril core, similar to recent observations of the core structure of A53T α-Syn fibrils (60). Alternatively, however, the variant may favor the formation of a fibril polymorph with more extensive rearrangements in the core structure, as observed for fibrils of the E46K variant (60).

It is also possible that the increased thermodynamic stability of H50Q α-Syn fibrils could contribute toward its increased rate of aggregation. The macroscopic aggregation rate, *k*_app_ (Fig. 2), is dependent on the microscopic rate of fibril elongation (61), which in turn depends on the free energy of the transition state, as depicted in Fig. 7. If the H50Q variant stabilizes this state (for example, if residue 50 is partially structured in the transition state), then this will also contribute toward the increased rate of aggregation, alongside the perturbations to the monomeric ensemble discussed above. Thus, it is important in the analysis of aggregation kinetics to consider both the fibrillar “products” of the reaction, as well as the monomeric “reactants.”

Finally, and importantly, the increased stability of fibrils formed from the H50Q variant corresponds to an order of magnitude reduction in the critical concentration, from 4.1 ± 0.1 μm for WT to 0.6 ± 0.1 μm for the variant. It is worth noting that the concentration of α-Syn in synaptosomes is ∼20 μm (62). It has been previously noted that the expression levels of proteins are finely tuned, together with their critical concentrations and aggregation rates, to avoid aggregation under normal physiological conditions (25–27). Indeed, many proteins, including α-Syn, are present *in vivo* at concentrations that exceed their critical levels and therefore exist in a metastable, or supersaturated, state in which only large kinetic barriers prevent their aggregation (25–27). In this context, the lowering of the critical concentration for the H50Q variant, particularly together with the acceleration of its aggregation, may tip this delicate balance toward aggregation and ultimately the onset of disease.

In conclusion, we have presented an analysis of the modulation of the aggregation process of the H50Q variant α-Syn by the WT form and of the features in their sequence and structure that are associated with their different aggregation behavior. Our results show that the changes in the ratio between the concentrations of the WT and H50Q α-Syn, in the secondary structure populations of the monomeric ensemble, and in the thermodynamic stability of the fibrils, are closely associated with an alteration of the aggregation behavior of this protein.

## Footnotes

4 The abbreviations used are:

α-Syn: α-Synuclein
ThT: thioflavin T
EM: electron microscopy
AFM: atomic force microscopy
BEST: band-selective excitation short transient.

## References

[1] Cookson M. R. (2009) α-Synuclein and neuronal cell death. Mol. Neurodegener. 4, 9 19193223 10.1186/1750-1326-4-9 PMC2646729

[2] Goedert M. (2001) α-Synuclein and neurodegenerative diseases. Nat. Rev. Neurosci. 2, 492–501 11433374 10.1038/35081564

[3] Farrer M. Maraganore D. M. Lockhart P. Singleton A. Lesnick T. G. de Andrade M. West A. de Silva R. Hardy J. Hernandez D. (2001) α-Synuclein gene haplotypes are associated with Parkinson's disease. Hum. Mol. Genet. 10, 1847–1851 11532993 10.1093/hmg/10.17.1847

[4] Krüger R. Kuhn W. Müller T. Woitalla D. Graeber M. Kösel S. Przuntek H. Epplen J. T. Schöls L. Riess O. (1998) Ala30Pro mutation in the gene encoding α-synuclein in Parkinson's disease. Nat. Genet. 18, 106–108 9462735 10.1038/ng0298-106

[5] Polymeropoulos M. H. Lavedan C. Leroy E. Ide S. E. Dehejia A. Dutra A. Pike B. Root H. Rubenstein J. Boyer R. Stenroos E. S. Chandrasekharappa S. Athanassiadou A. Papapetropoulos T. Johnson W. G. Lazzarini A. M. Duvoisin R. C. Di Iorio G. Golbe L. I. Nussbaum R. L. (1997) Mutation in the α-synuclein gene identified in families with Parkinson's disease. Science 276, 2045–2047 9197268 10.1126/science.276.5321.2045

[6] Singleton A. B. Farrer M. Johnson J. Singleton A. Hague S. Kachergus J. Hulihan M. Peuralinna T. Dutra A. Nussbaum R. Lincoln S. Crawley A. Hanson M. Maraganore D. Adler C. Cookson M. R. Muenter M. Baptista M. Miller D. Blancato J. Hardy J. Gwinn-Hardy K. (2003) α-Synuclein locus triplication causes Parkinson's disease. Science 302, 841 14593171 10.1126/science.1090278

[7] Zarranz J. J. Alegre J. Gómez-Esteban J. C. Lezcano E. Ros R. Ampuero I. Vidal L. Hoenicka J. Rodriguez O. Atarés B. Llorens V. Gomez Tortosa E. del Ser T. Muñoz D. G. de Yebenes J. G. (2004) The new mutation, E46K, of α-synuclein causes Parkinson and Lewy body dementia. Ann. Neurol. 55, 164–173 14755719 10.1002/ana.10795

[8] Conway K. A. Harper J. D. Lansbury P. T. (1998) Accelerated in vitro fibril formation by a mutant α-synuclein linked to early-onset Parkinson disease. Nat. Med. 4, 1318–1320 9809558 10.1038/3311

[9] Lashuel H. A. Hartley D. Petre B. M. Walz T. Lansbury P. T. Jr. (2002) Neurodegenerative disease: amyloid pores from pathogenic mutations. Nature 418, 291 12124613 10.1038/418291a

[10] Wood S. J. Wypych J. Steavenson S. Louis J. C. Citron M. Biere A. L. (1999) α-synuclein fibrillogenesis is nucleation-dependent. Implications for the pathogenesis of Parkinson's disease. J. Biol. Chem. 274, 19509–19512 10391881 10.1074/jbc.274.28.19509

[11] Spillantini M. G. Crowther R. A. Jakes R. Hasegawa M. Goedert M. (1998) α-Synuclein in filamentous inclusions of Lewy bodies from Parkinson's disease and dementia with lewy bodies. Proc. Natl. Acad. Sci. U.S.A. 95, 6469–6473 9600990 10.1073/pnas.95.11.6469 PMC27806

[12] Spillantini M. G. Schmidt M. L. Lee V. M. Trojanowski J. Q. Jakes R. Goedert M. (1997) α-Synuclein in Lewy bodies. Nature 388, 839–840 9278044 10.1038/42166

[13] Eliezer D. Kutluay E. Bussell R. Jr. Browne G. (2001) Conformational properties of α-synuclein in its free and lipid-associated states. J. Mol. Biol. 307, 1061–1073 11286556 10.1006/jmbi.2001.4538

[14] Fauvet B. Mbefo M. K. Fares M. B. Desobry C. Michael S. Ardah M. T. Tsika E. Coune P. Prudent M. Lion N. Eliezer D. Moore D. J. Schneider B. Aebischer P. El-Agnaf O. M. Masliah E. Lashuel H. A. (2012) α-Synuclein in central nervous system and from erythrocytes, mammalian cells, and Escherichia coli exists predominantly as disordered monomer. J. Biol. Chem. 287, 15345–15364 22315227 10.1074/jbc.M111.318949 PMC3346117

[15] Weinreb P. H. Zhen W. Poon A. W. Conway K. A. Lansbury P. T. Jr. (1996) NACP, a protein implicated in Alzheimer's disease and learning, is natively unfolded. Biochemistry 35, 13709–13715 8901511 10.1021/bi961799n

[16] Proukakis C. Dudzik C. G. Brier T. MacKay D. S. Cooper J. M. Millhauser G. L. Houlden H. Schapira A. H. (2013) A novel α-synuclein missense mutation in Parkinson disease. Neurology 80, 1062–1064 23427326 10.1212/WNL.0b013e31828727ba PMC3653201

[17] Appel-Cresswell S. Vilarino-Guell C. Encarnacion M. Sherman H. Yu I. Shah B. Weir D. Thompson C. Szu-Tu C. Trinh J. Aasly J. O. Rajput A. Rajput A. H. Jon Stoessl A. Farrer M. J. (2013) α-Synuclein p.H50Q, a novel pathogenic mutation for Parkinson's disease. Mov. Disord. 28, 811–813 23457019 10.1002/mds.25421

[18] Ghosh D. Mondal M. Mohite G. M. Singh P. K. Ranjan P. Anoop A. Ghosh S. Jha N. N. Kumar A. Maji S. K. (2013) The Parkinson's disease-associated H50Q mutation accelerates α-synuclein aggregation in vitro. Biochemistry 52, 6925–6927 24047453 10.1021/bi400999d

[19] Chi Y. C. Armstrong G. S. Jones D. N. Eisenmesser E. Z. Liu C. W. (2014) Residue histidine 50 plays a key role in protecting α-synuclein from aggregation at physiological pH. J. Biol. Chem. 289, 15474–15481 24742669 10.1074/jbc.M113.544049 PMC4140903

[20] Khalaf O. Fauvet B. Oueslati A. Dikiy I. Mahul-Mellier A. L. Ruggeri F. S. Mbefo M. K. Vercruysse F. Dietler G. Lee S. J. Eliezer D. Lashuel H. A. (2014) The H50Q mutation enhances α-synuclein aggregation, secretion and toxicity. J. Biol. Chem. 289, 21856–21876 24936070 10.1074/jbc.M114.553297 PMC4139205

[21] Rutherford N. J. Moore B. D. Golde T. E. Giasson B. I. (2014) Divergent effects of the H50Q and G51D SNCA mutations on the aggregation of α-synuclein. J. Neurochem. 131, 859–867 24984882 10.1111/jnc.12806

[22] Pawar A. P. Dubay K. F. Zurdo J. Chiti F. Vendruscolo M. Dobson C. M. (2005) Prediction of “aggregation-prone” and “aggregation-susceptible” regions in proteins associated with neurodegenerative diseases. J. Mol. Biol. 350, 379–392 15925383 10.1016/j.jmb.2005.04.016

[23] Tartaglia G. G. Pawar A. P. Campioni S. Dobson C. M. Chiti F. Vendruscolo M. (2008) Prediction of aggregation-prone regions in structured proteins. J. Mol. Biol. 380, 425–436 18514226 10.1016/j.jmb.2008.05.013

[24] Camilloni C. De Simone A. Vranken W. F. Vendruscolo M. (2012) Determination of secondary structure populations in disordered states of proteins using nuclear magnetic resonance chemical shifts. Biochemistry 51, 2224–2231 22360139 10.1021/bi3001825

[25] Baldwin A. J. Knowles T. P. Tartaglia G. G. Fitzpatrick A. W. Devlin G. L. Shammas S. L. Waudby C. A. Mossuto M. F. Meehan S. Gras S. L. Christodoulou J. Anthony-Cahill S. J. Barker P. D. Vendruscolo M. Dobson C. M. (2011) Metastability of native proteins and the phenomenon of amyloid formation. J. Am. Chem. Soc. 133, 14160–14163 21650202 10.1021/ja2017703

[26] Ciryam P. Tartaglia G. G. Morimoto R. I. Dobson C. M. Vendruscolo M. (2013) Widespread aggregation and neurodegenerative diseases are associated with supersaturated proteins. Cell Rep. 5, 781–790 24183671 10.1016/j.celrep.2013.09.043 PMC3883113

[27] Tartaglia G. G. Pechmann S. Dobson C. M. Vendruscolo M. (2007) Life on the edge: a link between gene expression levels and aggregation rates of human proteins. Trends Biochem. Sci. 32, 204–206 17419062 10.1016/j.tibs.2007.03.005

[28] DuBay K. F. Pawar A. P. Chiti F. Zurdo J. Dobson C. M. Vendruscolo M. (2004) Prediction of the absolute aggregation rates of amyloidogenic polypeptide chains. J. Mol. Biol. 341, 1317–1326 15302561 10.1016/j.jmb.2004.06.043

[29] Xiong H. Buckwalter B. L. Shieh H. M. Hecht M. H. (1995) Periodicity of polar and nonpolar amino acids is the major determinant of secondary structure in self-assembling oligomeric peptides. Proc. Natl. Acad. Sci. U.S.A. 92, 6349–6353 7603994 10.1073/pnas.92.14.6349 PMC41515

[30] Tartaglia G. G. Cavalli A. Vendruscolo M. (2007) Prediction of local structural stabilities of proteins from their amino acid sequences. Structure 15, 139–143 17292832 10.1016/j.str.2006.12.007

[31] Hoyer W. Antony T. Cherny D. Heim G. Jovin T. M. Subramaniam V. (2002) Dependence of α-synuclein aggregate morphology on solution conditions. J. Mol. Biol. 322, 383–393 12217698 10.1016/s0022-2836(02)00775-1

[32] Naiki H. Higuchi K. Hosokawa M. Takeda T. (1989) Fluorometric determination of amyloid fibrils in vitro using the fluorescent dye, thioflavin T1. Anal. Biochem. 177, 244–249 2729542 10.1016/0003-2697(89)90046-8

[33] Oosawa F. Kasai M. (1962) A theory of linear and helical aggregations of macromolecules. J. Mol. Biol. 4, 10–21 14482095 10.1016/s0022-2836(62)80112-0

[34] Narimoto T. Sakurai K. Okamoto A. Chatani E. Hoshino M. Hasegawa K. Naiki H. Goto Y. (2004) Conformational stability of amyloid fibrils of β2-microglobulin probed by guanidine-hydrochloride-induced unfolding. FEBS Lett. 576, 313–319 15498554 10.1016/j.febslet.2004.09.024

[35] Waudby C. A. Knowles T. P. Devlin G. L. Skepper J. N. Ecroyd H. Carver J. A. Welland M. E. Christodoulou J. Dobson C. M. Meehan S. (2010) The interaction of αB-crystallin with mature α-synuclein amyloid fibrils inhibits their elongation. Biophys. J. 98, 843–851 20197038 10.1016/j.bpj.2009.10.056 PMC2830463

[36] Sattler M. Schleucher J. Griesinger C. (1999) Heteronuclear multidimensional NMR experiments for the structure determination of proteins in solution employing pulsed field gradients. Prog. Nucl. Magn. Reson. Spectros. 34, 93–158

[37] Lescop E. Schanda P. Brutscher B. (2007) A set of BEST triple-resonance experiments for time-optimized protein resonance assignment. J. Magn. Reson. 187, 163–169 17468025 10.1016/j.jmr.2007.04.002

[38] Delaglio F. Grzesiek S. Vuister G. W. Zhu G. Pfeifer J. Bax A. (1995) NMRPipe: a multidimensional spectral processing system based on UNIX pipes. J. Biomol. NMR 6, 277–293 8520220 10.1007/BF00197809

[39] Vranken W. F. Boucher W. Stevens T. J. Fogh R. H. Pajon A. Llinas M. Ulrich E. L. Markley J. L. Ionides J. Laue E. D. (2005) The CCPN data model for NMR spectroscopy: development of a software pipeline. Proteins 59, 687–696 15815974 10.1002/prot.20449

[40] Wishart D. S. Bigam C. G. Yao J. Abildgaard F. Dyson H. J. Oldfield E. Markley J. L. Sykes B. D. (1995) ^1^H, ^13^C and ^15^N chemical shift referencing in biomolecular NMR. J. Biomol. NMR 6, 135–140 8589602 10.1007/BF00211777

[41] Ulrich E. L. Akutsu H. Doreleijers J. F. Harano Y. Ioannidis Y. E. Lin J. Livny M. Mading S. Maziuk D. Miller Z. Nakatani E. Schulte C. F. Tolmie D. E. Kent Wenger R. Yao H. Markley J. L. (2008) BioMagResBank. Nucleic Acids Res. 36, D402–D408 17984079 10.1093/nar/gkm957 PMC2238925

[42] Tartaglia G. G. Vendruscolo M. (2008) The Zyggregator method for predicting protein aggregation propensities. Chem. Soc. Rev. 37, 1395–1401 18568165 10.1039/b706784b

[43] Rose G. D. Geselowitz A. R. Lesser G. J. Lee R. H. Zehfus M. H. (1985) Hydrophobicity of amino acid residues in globular proteins. Science 229, 834–838 4023714 10.1126/science.4023714

[44] Janin J. (1979) Surface and inside volumes in globular proteins. Nature 277, 491–492 763335 10.1038/277491a0

[45] Stefanis L. (2012) α-Synuclein in Parkinson's disease. Cold Spring Harb. Perspect. Med. 2, a009399 22355802 10.1101/cshperspect.a009399 PMC3281589

[46] Kobayashi H. Krüger R. Markopoulou K. Wszolek Z. Chase B. Taka H. Mineki R. Murayama K. Riess O. Mizuno Y. Hattori N. (2003) Haploinsufficiency at the α-synuclein gene underlies phenotypic severity in familial Parkinson's disease. Brain 126, 32–42 12477695 10.1093/brain/awg010

[47] Voutsinas G. E. Stavrou E. F. Karousos G. Dasoula A. Papachatzopoulou A. Syrrou M. Verkerk A. J. van der Spek P. Patrinos G. P. Stöger R. Athanassiadou A. (2010) Allelic imbalance of expression and epigenetic regulation within the α-synuclein wild-type and p.Ala53Thr alleles in Parkinson disease. Hum. Mutat. 31, 685–691 20340137 10.1002/humu.21248

[48] Zanzoni A. Marchese D. Agostini F. Bolognesi B. Cirillo D. Botta-Orfila M. Livi C. M. Rodriguez-Mulero S. Tartaglia G. G. (2013) Principles of self-organization in biological pathways: a hypothesis on the autogenous association of α-synuclein. Nucleic Acids Res. 41, 9987–9998 24003031 10.1093/nar/gkt794 PMC3905859

[49] Croke R. L. Patil S. M. Quevreaux J. Kendall D. A. Alexandrescu A. T. (2011) NMR determination of pKa values in α-synuclein. Protein Sci. 20, 256–269 21280118 10.1002/pro.556 PMC3048411

[50] Dedmon M. M. Lindorff-Larsen K. Christodoulou J. Vendruscolo M. Dobson C. M. (2005) Mapping long-range interactions in α-synuclein using spin-label NMR and ensemble molecular dynamics simulations. J. Am. Chem. Soc. 127, 476–477 15643843 10.1021/ja044834j

[51] Bertoncini C. W. Jung Y. S. Fernandez C. O. Hoyer W. Griesinger C. Jovin T. M. Zweckstetter M. (2005) Release of long-range tertiary interactions potentiates aggregation of natively unstructured α-synuclein. Proc. Natl. Acad. Sci. U.S.A. 102, 1430–1435 15671169 10.1073/pnas.0407146102 PMC547830

[52] Waudby C. A. Mantle M. D. Cabrita L. D. Gladden L. F. Dobson C. M. Christodoulou J. (2012) Rapid distinction of intracellular and extracellular proteins using NMR diffusion measurements. J. Am. Chem. Soc. 134, 11312–11315 22694283 10.1021/ja304912c

[53] Waudby C. A. Camilloni C. Fitzpatrick A. W. Cabrita L. D. Dobson C. M. Vendruscolo M. Christodoulou J. (2013) In-cell NMR characterization of the secondary structure populations of a disordered conformation of α-synuclein within E. coli cells. PLoS One 8, e72286 23991082 10.1371/journal.pone.0072286 PMC3753296

[54] Camilloni C. Vendruscolo M. (2013) A relationship between the aggregation rates of α-synuclein variants and the β-sheet populations in their monomeric forms. J. Phys. Chem. B 117, 10737–107341 23941114 10.1021/jp405614j

[55] Kang L. Wu K. P. Vendruscolo M. Baum J. (2011) The A53T mutation is key in defining the differences in the aggregation kinetics of human and mouse α-synuclein. J. Am. Chem. Soc. 133, 13465–13470 21721555 10.1021/ja203979j PMC3205953

[56] Fitzpatrick A. W. Debelouchina G. T. Bayro M. J. Clare D. K. Caporini M. A. Bajaj V. S. Jaroniec C. P. Wang L. Ladizhansky V. Müller S. A. MacPhee C. E. Waudby C. A. Mott H. R. De Simone A. Knowles T. P. Saibil H. R. Vendruscolo M. Orlova E. V. Griffin R. G. Dobson C. M. (2013) Atomic structure and hierarchical assembly of a cross-β amyloid fibril. Proc. Natl. Acad. Sci. U.S.A. 110, 5468–5473 23513222 10.1073/pnas.1219476110 PMC3619355

[57] Comellas G. Lemkau L. R. Nieuwkoop A. J. Kloepper K. D. Ladror D. T. Ebisu R. Woods W. S. Lipton A. S. George J. M. Rienstra C. M. (2011) Structured regions of α-synuclein fibrils include the early-onset Parkinson's disease mutation sites. J. Mol. Biol. 411, 881–895 21718702 10.1016/j.jmb.2011.06.026 PMC3157309

[58] Heise H. Hoyer W. Becker S. Andronesi O. C. Riedel D. Baldus M. (2005) Molecular-level secondary structure, polymorphism, and dynamics of full-length α-synuclein fibrils studied by solid-state NMR. Proc. Natl. Acad. Sci. U.S.A. 102, 15871–15876 16247008 10.1073/pnas.0506109102 PMC1276071

[59] Vilar M. Chou H. T. Lührs T. Maji S. K. Riek-Loher D. Verel R. Manning G. Stahlberg H. Riek R. (2008) The fold of α-synuclein fibrils. Proc. Natl. Acad. Sci. U.S.A. 105, 8637–8642 18550842 10.1073/pnas.0712179105 PMC2438424

[60] Lemkau L. R. Comellas G. Lee S. W. Rikardsen L. K. Woods W. S. George J. M. Rienstra C. M. (2013) Site-specific perturbations of α-synuclein fibril structure by the Parkinson's disease associated mutations A53T and E46K. PLoS One 8, e49750 23505409 10.1371/journal.pone.0049750 PMC3591419

[61] Knowles T. P. Waudby C. A. Devlin G. L. Cohen S. I. Aguzzi A. Vendruscolo M. Terentjev E. M. Welland M. E. Dobson C. M. (2009) An analytical solution to the kinetics of breakable filament assembly. Science 326, 1533–1537 20007899 10.1126/science.1178250

[62] Wilhelm B. G. Mandad S. Truckenbrodt S. Kröhnert K. Schäfer C. Rammner B. Koo S. J. Classen G. A. Krauss M. Haucke V. Urlaub H. Rizzoli S. O. (2014) Composition of isolated synaptic boutons reveals the amounts of vesicle trafficking proteins. Science 344, 1023–1028 24876496 10.1126/science.1252884

